# Essential Oil of *Eucalyptus Gunnii* Hook. As a Novel Source of Antioxidant, Antimutagenic and Antibacterial Agents

**DOI:** 10.3390/molecules191119007

**Published:** 2014-11-18

**Authors:** Dušan Bugarin, Slavenko Grbović, Dejan Orčič, Dragana Mitić-Ćulafić, Jelena Knežević-Vukčević, Neda Mimica-Dukić

**Affiliations:** 1 Department of Chemistry, Biochemistry and Environmental Protection, Faculty of Sciences, University of Novi Sad, Trg Dositeja Obradovica 3, Novi Sad 21000, Serbia; E-Mails: dbugarin@t-com.me (D.B.); grbovicslavenko@gmail.com (S.G.); orcicdejan@gmail.com (D.O.); 2 Chair of Microbiology, Faculty of Biology, University of Belgrade, Studentski Trg 3/II, Belgrade 11000, Serbia; E-Mails: mdragana@bio.bg.ac.rs (D.M.-Ć.); jelenakv@bio.bg.ac.rs (J.K.-V.)

**Keywords:** *Eucalyptus gunnii*, essential oil, 1,8-cineole, antioxidative, antimutagenicity, antibacterial activity

## Abstract

The present study describes radical scavenging capacity (RSC), antimutagenic and antibacterial properties of the essential oil (*EO*) of the leaves of *Eucalyptus gunnii* Hook. (Southern Montenegro). Chemical composition was evaluated by gas chromatography-mass spectrometry (GC-MS). In oil, 1,8-cineole (67.8%) and α-pinene (14.12%) were the major compounds comprising almost 82% of total *EO*. *EO* exhibited moderate DPPH (2,2-diphenyl-1-picrylhydrazyl) scavenging activity, with IC_50_ value of 7.19 µL/mL. The antimutagenic properties were assayed against the spontaneous and *t*-BOOH-induced mutagenesis in *Escherichia coli* IC202 *oxyR* mutant strain, deficient in removing radical oxygen species (ROS). Reduction of the spontaneous mutagenesis in the presence of *E. gunnii* *EO* was only slight, up to 12% at the highest concentration tested. However, when the oxidative mutagen was used, *EO* displayed more significant reduction of mutagenesis (maximum 23%) in a concentration dependent manner. Antibacterial activity was tested against the selected strains from ATTC and NCIB collections: *Staphylococcus aureus*, *Staphylococcus epidermidis, Pseudomonas aeruginosa*, *Escherichia coli*, *Bacillus subtilis*, *Micrococcus flavus*, *Klebsiella pneumoniae*, and the two *Escherichia coli* strains from our laboratory collection (SY252 and IB112) using both the disk-diffusion and MIC assays. The greatest sensitivity was shown by *M. flavus*, *K. pneumoniae* and *E. coli lpc*A (MIC = 0.83 mg/mL), while the highest resistance was shown by *E. coli* (ATTC 25922) and *S. epidermidis*. This study represents the first report on chemical composition and biological activity of the *Eucalyptus gunnii* in the South Balkan region and beyond.

## 1. Introduction

Many aromatic plants and spices are well-known for their various beneficial effects on human health. Their use in phytotherapy is mostly related to different activities of their essential oils, such as antimicrobial, spasmolytic, carminative, antiviral, antimutagenic, anticarcinogenic, *etc.* [1,2]. Besides, many spices and essential oils are widely used in the food industry to improve flavor and organoleptic properties, but also to slow the process of deterioration of foodstuffs. The latter is mainly due to their antimicrobial and antioxidant activities [3,4].

The genus *Eucalyptus* (Myrtaceae) is one of the most important and most broadly planted genera. Although being native to Australia, more than 700 species are widely grown in many parts of the world. In fact, the eucalyptus species are one of the most-extensively planted pulpwood species. More than 300 species of this genus contain volatile oil in their leaves. However, less than 20 species have so far been exploited commercially for the production of essential oil rich in 1,8-cineole (>70%), which is essentially used in the pharmaceutical and cosmetic industries [5,6]. Used for centuries as a traditional Aboriginal herbal remedy, eucalyptus leaves and their essential oils find various applications in everyday life due to their antiseptic, anti-inflammatory and antipyretic properties [7,8]. Around 15 eucalyptus species grow in the Mediterranean region, fewer than 10 species having been introduced in the coastal area of Montenegro at the beginning of the 20th century. Among them *Eucaliptus camaldulensis* is the most widely planted. This species is used in the indigenous system of medicine to cure various human ailments, such as diarrhea, chronic dysentery, infection of upper respiratory tract, and certain skin diseases [9]. However, no information about the chemical composition and biological activity of the eucalyptus species in Montenegro had been published prior to initiating the study of *Eucaliptus camaldulensis* [10]. In pursuance of new sources of essential oils, we continued investigating the eucalyptus species throughout the South Balkan region. One of them is *Eucalyptus gunnii* Hook. (Myrtaceae), also known as cider gum or gunnii. This plant is endemic to Tasmania, occurring on the plains and slopes of the central plateau to around 1100 meters, with isolated occurrences south of Hobart [11]. *E. gunnii* is noted for exceptional cold tolerance for a eucalyptus (to −14 °C, exceptionally −20 °C for brief periods) and is now commonly planted as an ornamental tree across the British Isles and some parts of Western Europe [12]. Most of the recent research is focused on various physiological and molecular mechanisms, especially to cold resistance [13,14,15]. In addition, several papers are focused on lignin biosynthesis [16,17,18] as well as the possibility of cider gum exploitation as a source for bioenergy [19]. Only very few papers refer to the chemical composition and bioactivity of *E. gunnii* [20,21,22,23]. Plants belonging to the Myrtacae family are known to contain essential oil rich in oxygenated monoterpenes such as 1,8-cineole, linaloool, terpineol *etc.* Many of them have been qualified as natural antioxidant and antimutagenic agents [24,25]. Hence, it was reasonable to presume that *E. gunnii* and its volatile compounds could serve as a potential source of bioactive compounds. Therefore, the present study was undertaken to investigate essential oil composition, as well as antioxidant, antimutagenic and antibacterial properties of *E. gunni* from Montenegro.

## 2. Results and Discussion

### 2.1. Essential Oil Composition

The oil yields in the examined *E. gunnii* leaves were 1.76 g/100 g dried leaves, respectively.

Thirty-one compounds, which represent 99.22% of the total *EO*, were identified. In the oil sample, the dominant were oxygenated monoterpenes, which accounted for 77.41%. The major compounds were 1,8-cineole (67.8%) and α-pinene (14.12%), respectively (Table 1).

**Table 1:** Chemical composition (%) of essential oil of *E. gunnii*.

No.	Compound	RI ^a^	*EO* (%)
**1**	α-thujene	927	tr
**2**	α-pinene	934	14.12
**3**	Sabinene	974	tr ^b^
**4**	2-β-pinene	978	0.38
**5**	β-myrcene	992	tr
**6**	α-phellandrene	1006	tr
**7**	α-terpinene	1018	tr
**8**	p-cymene	1026	0.69
**9**	β-phellandrene	1030	3.92
**10**	1,8-cineole	1033	67.80
**11**	γ-terpinene	1060	tr
**12**	α-terpinolene	1090	tr
**13**	Linalool	1101	tr
**14**	Cis-p-menth-2-en-1-ol	1126	tr
**15**	Trans-pinocarveol	1144	2.49
**16**	Pinocarvone	1168	0.48
**17**	4-terpineol	1182	0.34
**18**	P-cymen-7-ol	1188	tr
**19**	Cryptone	1190	0.45
**20**	α-terpineol	1194	2.08
**21**	Myrtenal	1199	tr
**22**	Cuminaldehyde	1246	tr
**23**	Phellandral	1281	tr
**24**	Cumyl alcohol	1294	tr
**25**	Thymol	1302	tr
**26**	α-terpinyil acetate	1355	3.27
**27**	Aromadendrene	1451	0.71
**28**	Allo-aromadendrene	1474	0.32
**29**	Byciclogermacrene	1509	0.54
**30**	Spathulenol	1593	0.43
**31**	Viridiflorol	1606	0.53
	**Identified Compounds**		99.22
	Monoterpene hydrocarbons		19.26
	Oxygenated monoterpenes		77.41
	Sesquiterpene hydrocarbons		1.58
	Oxygenated sesquiterpenes		0.96

^a^ Retention indices relative to C_9_-C_24_ *n*-alkanes in the HP-5MS column; ^b^ tr-abundance in essential oil below 0.2%.

When searching for literature data, we found only a few references dealing with *E. gunnii* essential oil [20,21,22,23]. One of the most comprehensive is the study undertaken by Ellaissi *et al*. [20] who reported on volatile oil composition of 13 eucalyptus species growing in Tunisia. According to the principal compound analysis (PCA) and Hierarchical Cluster Analysis (HCA), the *Eucalyptus gunnii* was grouped into the section with high content of oxygenated sesquiterpenes, in particular spathulenol, globulol and viridiflorol, and low content of 1,8-cineole. Because of its very high ratio of 1,8-cineole, we found our sample similar to species *E. macarthurii* and *E.cinerea* that had 54.8% and 70.4% of 1,8-cinole, respectively. In addition, the same authors emphasized high variation of volatile compounds in particular species of different geographical origin. Unlike the results mentioned, our results are very similar to those reported by Li *et al*. [26]. They examined the volatile oil variation in plants of the eucalyptus genera, divided in two subgenera *Monocalyptus* and *Symphyomyrtus*, growing in Tasmania [26,27]. It was reported that species belonging to the *Symphyomyrtus* subgenus characteristically have a high content of 1,8-cineole and α-pinene. In the essential oil of *E. gunnii*, they found a high content of 1,8-cineole (38.1%), α-pinene (16.1%), limonene (3.9%) and p-cymene (7.4%) among monoterpenes and the highest ratio of viridiflorol (9.7%) and β-eudesmol (5.8%), among the sesquiterpenes. Concerning the oil yield, our results (1.76%) are in accordance with those obtained by Li at al. (2.8%), but do not match those obtained by Ellaissi *et al*. (0.5%) [20]. All this indicates that *E. gunnii* plants growing in Montenegro are similar to those in Tasmania, which is their natural habitat, but quite different from the plants in Tunisia. It is important to point out that because of its essential oil composition *E. gunnii* in Montenegro could be used as an important source of 1,8-cinole, substance with various uses in industry.

### 2.2. Antioxidant Activity

The results from the DPPH test for *E. gunnii EO* are presented in Table 2. The essential oils were able to reduce the stable radical DPPH to the yellow-colored DPPH-H reaching 50% of reduction with an IC_50_ of 7.19 µL/mL.

**Table 2:** DPPH-radical scavenging capacity (RCS) of *Eucalyptus gunnii* essential oil (*EO*) and BHA, BHT and PG as positive controls.

*EO*	Volume (μL)	Concentration (μL/mL)	Inhibition (%)	IC_50_ (μL/mL)
***EO***	5.0	1.25	12.150	
	10.0	2.50	20.561	
	20.0	5.00	40.498	
	40.0	10.0	60.436	7.19 ± 0.81
	60.0	15.0	75.389	
**Standard antioxidants**				**IC_50_ (μg/mL)**
BHT				8.62 ± 0.50
BHA				3.09 ± 0.36
PG				0.42 ± 0.055

Values are the means of ±SD of three replications.

The radical scavenging capacity of *E. gunnii* *EO* was significantly lower compared to the commercial antioxidants (BHT, BHA and PG). In several previous studies, we showed that the essential oil antioxidant activity is closely related to the content of aromatic compounds, such as *p*-cymene, eugenol, thymol, carvacrol, *etc.* [28,29,30] Taking into account that non-aromatic compounds, such as α-pinene and 1,8-cineole comprise almost 82% of *E. gunnii* *EO*, somewhat lower DPPH activity was expected. Furthermore, in our previous studies we showed that in many cases essential oil exhibited lower DPPH scavenger activities than MeOH (EtOH) extracts of the same plant species [3,4]. Thus, we found that MeOH extract of *E. gunnii* shows very high ROS capacity towards DPPH, superoxide anion and hydroxyl radicals [31]. These results are in accordance with those obtained by Guimaraes *et al.* [23]. Besides, it has to be kept in mind that one test does not suffice to valorize the antioxidant efficiency of plant products, especially of such complex mixtures as essential oils [32,33].

### 2.3. Antimutagenic Activity

The antimutagenic effect of the *E. gunnii* oil against the spontaneous and *t*-BOOH-induced mutagenesis was tested in *E. coli* IC202 *oxyR*, a bacterial strain deficient in removing ROS. Since the strain carries the *trpE65* mutation, we followed the effect by monitoring the percentage of Trp^+^ revertants. Deficiency in removing ROS is a consequence of mutation in the *oxyR* gene, leading to deficiency in the OxyR function. The OxyR protein is a redox-sensitive transcriptional activator of genes encoding antioxidant enzymes: catalase, alkyl hydroperoxide reductase and glutathione reductase, which are produced by the cells in response to oxidative stress [34]. Therefore, the IC202 strain is highly sensitive to oxidative DNA damage [35]. The results of antimutagenicity testing are shown in Table 3.

The *EO* concentrations applied (0.05–0.15 μL/p) were lower than the maximum non-toxic concentration (0.2 μL/p), which was previously determined by a toxicity assay. The inhibition of the spontaneous mutagenesis in the presence of the *E. gunnii* *EO* was only slight, reaching 12% at the highest concentration tested. When the oxidative mutagen was used, *EO* displayed a higher reduction of mutagenesis. Even at the lowest concentration (0.05 μL/p), a significant reduction of mutagenesis was obtained (18%), which slightly increased and achieved the maximum at the highest concentration applied (23%). Our results are in agreement with those previously published [25,34,35], where the essential oils and some of their constituents were shown to possess a considerable antimutagenic activity. Vuković-Gačić *et al.* [34] explored the antimutagenic activity of sage oil and its dominant monoterpenes: 1,8-cineole, limonene, thujone, and camphor towards UV-induced mutagenesis in *E. coli*. They found out that oxygenated monoterpenes expressed a significant antimutagenic potential, whereas limonene failed to show any antimutagenicity.

**Table 3:** Effect of *Eucalyptus gunnii* essential oil (*EO*) on spontaneous and *t*-BOOH-induced mutagenesis in *E. coli* IC202.

*EO* (μL/p)	−*t*-BOOH		+*t*-BOOH ^1^	
	Revert/p ^2^	*M* (%) ^3^	Revert/p ^2^	*M* (%) ^3^
0 ^4^	97 ± 8	100	190 ± 10	100
0.05	92 ± 33	95	156 ± 17 *	82
0.075	101 ± 19	104	148 ± 22 *	78
0.10	88 ± 18	91	148 ± 25 *	78
0.15	85 ± 15	88	147 ± 13 *	77

^1^ *t*-BOOH induced mutagenesis, applied concentration 25 µg/p. These values are an average of duplicate samples from three independent experiments; ^2^ Trp/mL = Trp^+^ revertants/p × 10; ^3^ *M—*mutagenesis (%M = (Nt/Nc) × 100. ^4^ *n*-hexane—solvent control Nt, sample with *EO*; Nc control sample (*n*-hexane); * (*p* < 0.05).

Even more relevant are the findings of Mitić-Ćulafić *et al.* [25] who established a considerable protective effect of linalool, myrcene and eucalyptol (1,8-cineole) against *t*-BOOH-induced genotoxicity in *E. coli* and cultured human cells. In our previous study [24] we showed that *Myrtus communis* *EO* reduced the *t*-BOOH induced mutagenticity by 28%, at the highest concentration applied (0.15 μL/p). The inhibition of mutagenesis was similar to one obtained here by *E. gunnii* oil (23%). Bearing in mind that the bacterial cells were treated with antioxidant (*EO*) and mutagen (*t*-BOOH) simultaneously, we can presume that the reduction of mutagenesis is related to either scavenging of ROS or direct interaction with *t*-BOOH. The available data on antimutagenic properties of the eucalyptus plants are limited, so we have reason to believe that this is one of the first reports on the antigenotoxic effect of *E. gunnii*. The results obtained indicate that *E. gunnii* essential oil has a substantial protective activity against oxidant-induced mutagenesis, which is predominantly mediated by their radical scavenging activity.

### 2.4. Antibacterial Activity

In a preliminary experiment, we screened the effect of essential oil of *E. gunnii* against *Staphylococcus aureus* ATCC25923, *Staphylococcus epidermidis* ATCC12228, *Pseudomonas aeruginosa* ATCC27853, *Escherichia coli* ATCC25922, *Bacillus subtilis* ATCC10774, *Micrococcus flavus* ATCC10240, *Klebsiella pneumoniae* NCIB9111, and *Escherichia coli* SY252 and IB112 in disc-diffusion assay (Table 4).

**Table 4:** Antibacterial activity of *E. gunnii* *EO* tested by disc-diffusion method.

Bacterial Strains	*EO* Concentrations (µL/disc)					Antibiotics ^b^
	3.32	1.66	0.83	0.41	*n*-hexane	
*S. epidermidis* ATCC12228	25 ± 1.0 ^a^	20 ± 1.5	(12 ± 1.0)	(15 ± 1.0)	0	Gentamyicin 35 ± 0.25
*S. aureus* ATCC25923	(26 ± 1.7)	(23 ± 2.0)	0	0	0	Streptomycin 22 ± 0.31
*B. subtilis* ATCC10774	29 ± 2.1	20 ± 1.0	(17 ± 1)	0	0	Streptomycin 27 ± 0.28
*M. flavus* ATCC10240	(26 ± 1.5)	(19 ± 1.0)	(18 ± 0.5)	0	0	Bacitracin 33 ± 0.15
*E. coli* ATCC25922	(29 ± 1.5)	(18 ± 0.5)	(20 ± 1.0)	0	0	Streptomycin 20 ± 0.43
*P. aeruginosa* ATCC27853	(24 ± 2.6)	(17 ± 1.7)	(13 ± 1.5)	0	0	Streptomycin 20 ± 0.19
*K. pneumoniae* NCIB9111	(26 ± 1.0)	(21 ± 2.0)	(15 ± 0.6)	(11 ± 1.2)	0	Chloramphenicol 19 ± 0.43
*E. coli* SY252	(31 ± 3.6)	(25 ± 1.2)	(18 ± 1.0)	0	0	Streptomicyn 23 ± 0.18
*E. coli* IB112 (*lpcA*)	(30 ± 1.7)	(20 ± 0.6)	(18 ± 1.2)	0	0	Streptomycin 23 ± 0.35

^a^ Diameter of inhibition zone (mm); 0—no growth inhibition zone; brackets indicate incomplete inhibition of growth; ^b^ chloramphenicol (30 μg/disc), streptomycin (100 μg/disc), bacitracin (0.04 IU/disc) and gentamycin (40 μg/disc).

The results of disc-diffusion testing demonstrated that *EO* showed antimicrobil activity only on two Gram-positive bacteria *S. epidermidis* and *B. subtilis* in concentration higher than 1.66 μL/mL. The other Gram-positive and Gram-negative bacteria were mostly resistant and showed only low sensitivity that was manifested by the presence of an enlightened rather than a clear zone around the discs, even at the highest concentration applied. Obtained results are in agreement with those reported by Elaissi *et al.* [22] and Luqman *et al.* [36].

However, it should be noted that the disc-diffusion method is limited by the hydrophobic nature of most essential oils, which prevents their uniform diffusion through the agar medium. Therefore, the majority of authors prefer liquid medium methods: minimal inhibition concentration (MIC) and minimal bacterial concentration (MBC) [37]. In Table 5 we present the results of the MIC test. The results show that the highest sensitivity to *E. gunni* essential oil was demonstrated by *M. flavus*, *K. pneumoniae*, and *E. coli lpc*A, where the MIC was 0.83 mg/mL, while the highest resistance was shown by *S. aureus*, *B. subtilis*, *E. coli* SY252, where the MIC was 1.66 µL/mL, and *S. epidermidis*, where the MIC was 3.32 mg/mL. The total resistance to the applied concentrations of *EO* was manifested by *E. coli* ATCC25922, where the MIC was not determined even with the highest amount of the *EO* applied. Duarte *et al.* [38] proposed a classification to be applied to the extracts based on MIC values; this author considers an MIC of up to 500 μg/mL as strong inhibitors, MIC between 600 and 1500 μg/mL as moderate inhibitors, and MIC above 1600 μg/mL as weak inhibitors. As for the results obtained, *E. gunni* essential oil can be classified as a moderate antibacterial agent against *M. flavus*, *K. pneumoniae*, and *E. coli lpc*A, and a weak one against the other bacteria tested.

Slight antibacterial activity of *E. gunnii* essential oil can be explained by the fact that the dominant component in *EO*, 1,8-cineole, does not demonstrate antibacterial activity [39]. In addition, Mitić *et al.* [40], in their study on antimicrobial efficiency of sage oil, reported a very low antibacterial activity of 1,8-cineole against *S. aureus*, *B. subtilis*, and *E. coli* SY252, but a very strong one of α-thujone, especially on *E. coli* SY252. However, it is more likely that the biological effects are the result of synergism of all the molecules contained in an essential oil.

**Table 5:** MIC values of essential oil (*EO*) of *E. gunnii* and streptomycin as reference antibiotic.

Bacterial Strain	MIC (mg/mL) *EO*	MIC (μg/mL) Streptomycin
*S. epidermidis* ATCC12228	3.32	25
*S. aureus* ATCC25923	1.66	50
*M. flavus* ATCC10240	0.83	25
*B. subtilis* ATCC10774	1.66	10
*K. pneumoniae* NCIB9111	0.83	50
*E. coli* ATTC 25922	not identified	12.5
*E. coli* SY252	1.66	25
*E. coli* IB112 (*lpcA*)	0.83	12.5

## 3. Experimental Section

### 3.1. Plant Material and Chemicals

Plant material: The leaves of *E. gunnii* plants were collected in August 2006 from Podgorica, Montenegro. The Voucher specimens were prepared and identified by Goran Anačkov, PhD, and deposited at the Herbarium of the Department of Biology and Ecology (No 2-1818; No 2-1823, BUNS Herbarium), University of Novi Sad, Faculty of Sciences, Novi Sad, Serbia.

Chemicals: 1,1-Diphenyl-2-(2,4,6-trinitrophenyl) hydrazine (syn: 2,2-diphenyl-1-picrylhydrazyl, DPPH), 2-thiobarbituric acid (TBA), sulfanilamide, *tert*-butylated hydroxytoluene (BHT) were obtained from FlukaChemie GmbH (Buchs, Switzerland). Propyl galate, PG (Propyl 3,4,5-trihydroxybenzoate) and *tert*-Butyl-4-hydroxyanisole (BHA) were obtained by ICN Biochemical (Clevelend, OH, USA). Trichloroacetic acid was purchased from Lach-Ners.r.o. (Neratovice, Czech Republic), *n*-hexane (Merck, Darmstadt, Germany); *t*-butyl hydroperoxide (*t*-BOOH, Aldrich, CAS No. 75-91-2).

### 3.2. Essential Oil Isolation and Analysis

Air-dried leaves were submitted to hydrodistillation according to Eur. Pharm. 4 [41], using *n*-hexane as a collecting solvent. The solvent was removed under vacuum, and the quantities of the essential oils were gravimetrically determined.

*GC-MS analysis of the essential oil*: Essential oil analysis was done as described in our previous study [22]. In brief: qualitative analysis of the essential oils was performed by gas chromatography-mass spectrometry (GC-MS). An Agilent Technologies (6890N-5975C) system was used, with data acquisition parameters as follows: carrier gas—He purity 99.999%), flow 1.0 mL/min, constant flow mode; injection volume 0.2 μL (split 50:1), inlet temperature 250 °C; Agilent Technologies HP-5MS column (30 m × 0.25 mm × 0.25 μm), temperature program: 50 °C for 1 min, 5 °C/min to 100 °C/min, 9 °C/min to 200 °C, hold 7.89 min; transfer line temperature 280 °C; electron ionization, electron energy 70 eV, Scan mode, (*m/z* range 35–400), quadrupole temperature 150 °C, source temperature 230 °C. Acquired data were analyzed by Agilent Technologies MSD ChemStation software in conjunction with AMDIS (Automated Mass Spectral Deconvolution and Identification System) and NIST MS Search software. Two different mass spectra libraries were used for the mass spectra identification: the Wiley Registry of Mass Spectral Data 7th Edition (338,000 spectra, 289,000 unique compounds) [42], and the NIST/EPA/NIH Mass Spectral Library 05 with 190,825 spectra and 163,198 unique compounds (*NIST/EPA/NIH Mass Spectral Library with Search Program*, 2005) and confirmed by comparison of Kovats retention indices (KI) with the literature data [43]. Diesel oil, consisting of a mixture of C_8_-C_28_ *n*-alkanes corresponding to 800–2800 KI was used as a standard for determination of retention indices. Relative amounts of the components, expressed in percentages, were calculated by normalization measurement accounting to peak area in total ion chromatogram.

### 3.3. Antioxidant Activity Assay

*Free radical scavenging capacity (RSC):* The RSC was evaluated by measuring the scavenging activity of *EO* samples on the stable DPPH radical. The DPPH assay was performed as previously described [24]. Various amounts of the essential oil samples (5, 10, 20, 40 and 60 mg) were mixed with 1 mL of 90 µM DPPH solution and made up to a final volume of 4 mL with 95% MeOH. The final concentrations of oils are reported in Table 2. In the control, the *EO* was substituted with a similar amount of solvent. The well-known artificial synthetic antioxidants BHT, BHA and PG were used as a positive control. Monitoring was continued for 70 min until the reaction reached a plateau. For each sample four replicates were recorded. The disappearance of DPPH was read spectrophotometrically at 515 nm using a Beckman DU-65 spectrophotometer. The percent of RSC was calculated using the following equation:

RSC (%) = 100 − (Ablank − Asample/Ablank)

The extract concentration providing 50% of radicals scavenging activity (IC_50_) was calculated by interpolation from the graph of RSC percentage against extract concentration.

### 3.4. Antimutagenic Activity

*Bacterial Strains and Media:* The tester strain used in this study was *E. coli* WP2 mutant IC202 *trpE65 oxyR*/pKM101 [35]. The media used were: (i) LB medium (5 g yeast-extract, 10 g bacto-tryptone, 5 g NaCl, 1000 mL distilled water; (ii) LA medium (the same as LB but 15 g Difco agar was added); (iii) Solid minimal E4 medium containing 15 g Difco agar, 4 g glucose and 20 mL Vogel-Bonner E buffer; (iv) ET4 plates (minimal medium, supplemented with 0.5 mg/L tryptophan); (v) top agar containing 6 g Difco agar and 5 g NaCl (per liter of distilled water).

#### 3.4.1. Toxicity Assay

Toxicity of *EO* to IC202 strain was determined in order to find the non-toxic concentrations for antimutagenicity assay. Overnight culture of IC202 in LB (0.1 mL) was added to 3 mL of molten top agar (45 °C) and poured onto LA plates. Different dilutions of *EO* were added on sterile filter paper discs (6 mm in diameter), placed onto the inoculated surface, and incubated for 24 h at 37 °C. A clear zone of growth inhibition around the disc indicated a toxic effect.

#### 3.4.2. Bacterial WP2 Antimutagenicity Assay

The overnight culture of *E. coli* IC202 was grown in LB medium at 37 °C. The antimutagenicity assay was performed by mixing 0.1 mL of fresh overnight cultures of bacteria, 0.1 mL of buffer dilution of *t*-BOOH (final concentration 25 µg/plate), appropriate concentration of *EO* (0.05, 0.75, 0.1 and 0.15 µL/plate) diluted in hexane, and 3 mL of molten top agar (45 °C). The mixture was poured onto ET4 plates [35]. The number of Trp^+^ revertants was scored after incubation for 48 h at 37 °C. Simultaneously, the influence of *EO* on spontaneous mutagenesis was examined. Plating on E4 (minimal medium, without tryptophan) was used to evaluate the number of pre-plating mutants originating during the overnight growth. The overnight cultures with a low number of pre-existing revertants (˂15 revertants per plate) were used for experiments. In addition, the effect of *EO* on viability of the cells untreated and treated with *t*-BOOH was tested by plating the appropriately diluted overnight cultures on LA plates [44]. Hexane was used as a negative control. The experiments were carried out at least three times, each with two replicates. The percentage of inhibition of mutagenesis (%I) was calculated as described in our previous paper [24].

### 3.5. Antibacterial Activity

#### 3.5.1. Bacteria and Media

The following bacterial strains were used: Staphylococcus aureus ATCC25923, Staphylococcus epidermidis ATCC12228, Pseudomonas aeruginosa ATCC27853, Escherichia coli ATCC25922, Bacillus subtilis ATCC10774, Micrococcus flavus ATCC10240, Klebsiella pneumoniae NCIB9111 and Escherichia coli SY252 and IB112 strains from our laboratory collection. IB112 strain is characterized by increased permeability due to lpcA mutation. Bacteria were cultivated at 37 °C in Luria broth (LB) (yeast extract 5 g, bacto-tryptone 10 g, NaCl 5 g, distilled water 1 L), or Mueller Hinton Broth (MHB) from Oxoid (Basingstoke, UK). Luria Agar (LA, LB plus 15 g agar) and Mueller Hinton Agar (MHA) from Oxoid were used as solid media. The essential oils were dissolved in ethanol (1/10) and applied in different concentrations.

#### 3.5.2. Disc-Diffusion Assay

The disc-diffusion assay was applied to determine the growth inhibition of bacteria by *EO* [45]. Overnight bacterial cultures (100 μL) were spread onto MHA. *EO* was applied to 10 mm discs (Whatman paper No.1). After 24 h of incubation at 37 °C, the diameter of growth inhibition zones was measured. Ethanol was used as a negative control and antibiotics: chloramphenicol (30 μg/disc), streptomycin (100 μg/disc), bacitracin (0.04 IU/disc) and gentamycin (40 μg/disc) as positive controls.

#### 3.5.3 MIC Determination

The broth dilution test was performed in test tubes. In two-fold serial dilutions of EO, a standardized suspension (McFarland turbidity standard) of test bacteria (100 μL) was added to obtain a final concentration of 5 × 10^5^ CFU/mL. A growth control tube and sterility control tube were used in each test. After the overnight incubation at 37C, the MIC was determined visually as the lowest concentration that inhibits growth, evidenced by the absence of turbidity [45]. Differences of more than two steps of dilutions were considered significant [46].

### 3.6. Statistical Analysis

The t-test was employed for statistical analysis. The significance was tested at the *p* < 0.05 level. Values in Tables are averages with standard errors.

## 4. Conclusions

This paper describes a study of the chemical composition, antioxidant, antimutagenic and antibacterial properties of the essential oil obtained from the leaves of *E. gunnii* growing in Montenegro. The study proves that *E. gunni* is very rich in essential oil, with an especially high amount of 1,8-cineole, and could be regarded as a potential source for the food, pharmaceutical and cosmetic industries. Furthermore, *E. gunnii* *EO* showed a considerable antimutagenic potential in a non-toxic concentration range. In addition, the antibacterial assays demonstrated moderate activity against *M. flavus*, *K. pneumoniae* and *E. coli lpc*A. Taken altogether, the results presented here support the potential use of the essential oil from *Eucalyptus gunnii*, but at the same time indicate the need for additional investigations to valorize its application as health-beneficial phytochemical and new remedy. In conclusion, it should be pointed out that in the present study a chemical and biological examination of *Eucaliptus gunni* from this part of Europe was undertaken for the first time.
